# The Use of Triphenyl Phosphonium Cation Enhances the Mitochondrial Antiplatelet Effect of the Compound Magnolol

**DOI:** 10.3390/ph16020210

**Published:** 2023-01-30

**Authors:** Francisca Tellería, Santiago Mansilla, Diego Méndez, Magdalena Sepúlveda, Ramiro Araya-Maturana, Laura Castro, Andrés Trostchansky, Eduardo Fuentes

**Affiliations:** 1 MIBI: Interdisciplinary Group on Mitochondrial Targeting and Bioenergetics, Department of Clinical Biochemistry and Immunohematology, Thrombosis Research Center, Medical Technology School, Faculty of Health Sciences, Universidad de Talca, Talca 3480094, Chile; 2 Departamento de Métodos Cuantitativos and Centro de Investigaciones Biomédicas (CEINBIO), Facultad de Medicina, Universidad de la República, Montevideo 11800, Uruguay; 3 MIBI: Interdisciplinary Group on Mitochondrial Targeting and Bioenergetics, Instituto de Química de Recursos Naturales, Universidad de Talca, Talca 3460000, Chile; 4 Departamento de Bioquímica and Centro de Investigaciones Biomédicas (CEINBIO), Facultad de Medicina, Universidad de la República, Montevideo 11800, Uruguay

**Keywords:** platelet, mitochondria, magnolol, triphenylphosphonium, respiration

## Abstract

Although platelets are anucleated cells, they have fully functional mitochondria, and currently, it is known that several processes that occur in the platelet require the action of mitochondria. There are plenty of mitochondrial-targeted compounds described in the literature related to cancer, however, only a small number of studies have approached their interaction with platelet mitochondria and/or their effects on platelet activity. Recent studies have shown that magnolia extract and mitochondria-targeted magnolol can inhibit mitochondrial respiration and cell proliferation in melanoma and oral cancer cells, respectively, and they can also induce ROS and mitophagy. In this study, the effect of triphenylphosphonium cation, linked by alkyl chains of different lengths, to the organic compound magnolol on human-washed platelets was evaluated. We demonstrated that the addition of triphenylphosphonium by a four-carbon linker to magnolol (MGN4) considerably enhanced the Magnolol antiplatelet effect by a 3-fold decrease in the IC_50_. Additionally, platelets exposed to MGN4 5 µM showed several differences from the control including increased basal respiration, collagen-induced respiration, ATP-independent respiration, and reduced ATP-dependent respiration and non-mitochondrial respiration.

## 1. Introduction

Cardiovascular diseases correspond to one of the main causes of death [1] and disability in adults worldwide, and their incidence also begins to increase with age [2]. Among the main risk factors due to medical causes that predispose a person to suffer from this condition are hypercholesterolemia, hypertension, and diabetes mellitus [2]. Other risk factors such as diet, sedentary lifestyle, and alcohol and tobacco consumption, predispose one to the development of cardiovascular diseases [3].

Platelets correspond to small blood cells (2–4 µm) anucleated [4] that originate from the fragmentation of the cytoplasm of megakaryocytes in the bone marrow and remain in circulation for 7–10 days [5]. In a healthy adult, the concentration of platelets ranges between 150,000–400,000 platelets/µL [6], the count of which varies with age and the state of health of the person. They play an important role in managing hemostasis [5] and are the main actors involved when a blood vessel is damaged to prevent blood loss [7]. Although their role is key, when there is deregulation in their activation, their function goes from being beneficial to being detrimental to the organism [8]. Although platelets are anucleated, they have fully functional mitochondria [9], where there are around five to eight mitochondria for each healthy platelet [10]. As above-mentioned, platelets lack a nucleus, for which the duration of their life in circulation is determined by their mitochondria [10]. Due to this, any damage or mitochondrial dysfunction affects both the life of the platelets and their function.

The mitochondria correspond to an organelle that regulates multiple functions and also plays an important role in programmed cell death [11]. In platelets, both oxidative phosphorylation and glycolysis play important roles in platelet energy production in the basal state, while half of the mitochondrial function is dedicated to ATP production [12]. Several processes that occur in the platelet require the action of mitochondria such as the regulation of calcium homeostasis [10] and activated platelets, which exhibit a phenotype inclined toward the glycolytic pathway [13]. On the other hand, platelets have also shown “metabolic flexibility” and can use glycolysis or fatty acid catabolism to replace oxidative phosphorylation (mitochondrial ATP production) in adverse situations such as hypoxia or the use of mitochondrial inhibitory agents [14,15], which helps them meet the energy demand needed for their functions [10].

Increasing evidence has shown that mitochondria play a crucial role in the development and continuity of several diseases including cancer and cardiovascular disease, therefore, the use of different vehicles has been studied for the mitochondrial delivery of small molecules of interest [16,17,18,19,20]. Given the negative membrane potential of the mitochondrial inner membrane, positively charged compounds can accumulate in the mitochondrial matrix against their concentration gradient, making them effective delivery vehicles [20,21,22]. Such is the case of the triphenylphosphonium cation (TPP+), which has proven to be effective when directing mitochondria-targeted compounds and, depending on the length of the linker alkyl chain (typically *n* = 2–10), certain factors such as the lipophilicity, cellular uptake, cytotoxicity, and site of mitochondrial sequestration (matrix or membrane) can be altered [18,19,20,21,22,23].

There are plenty of mitochondrial-targeted compounds described in the literature related to cancer, however, only a small number of studies have approached their interaction with platelet mitochondria and/or their effects on platelet activity. Some of these compounds are salvianolic acid, xanthohumol, mito-TEMPO, mitoQ, and metformin, with effects ranging from antiplatelet and anti-thrombosis to a protective effect against oxidative damage or stress, among others [24,25,26,27,28]. Recent studies have shown that mitochondria-targeted magnolol and magnolia extract can inhibit mitochondrial respiration and cell proliferation in melanoma and oral cancer cells respectively, and it can also induce ROS and mitophagy [29,30].

Magnolia extract (ME), obtained from *Magnolia officinalis* and other species of the Magnoliaceae family, is a traditional herbal medicine that has been used for thousands of years in East Asia for its capacity to treat various diseases [31]. It was reported that ME contains several bioactive compounds such as magnolol (MGN, 5,5′-diallyl-2,2′-dihydroxybiphenyl), one of its three major compounds, which has been shown to have several properties such as anti-cancer, anti-platelets, anti-oxidant, anti-inflammatory, antibiotic, and antispastic effects, among others [32,33,34,35,36,37,38,39,40,41]. In this study, the effect of linking the triphenylphosphonium cation by alkyl chains of different lengths to the organic compound magnolol on human-washed platelets was evaluated.

## 2. Results

### 2.1. Cytotoxic Effect of Compounds

**Figure 1:**
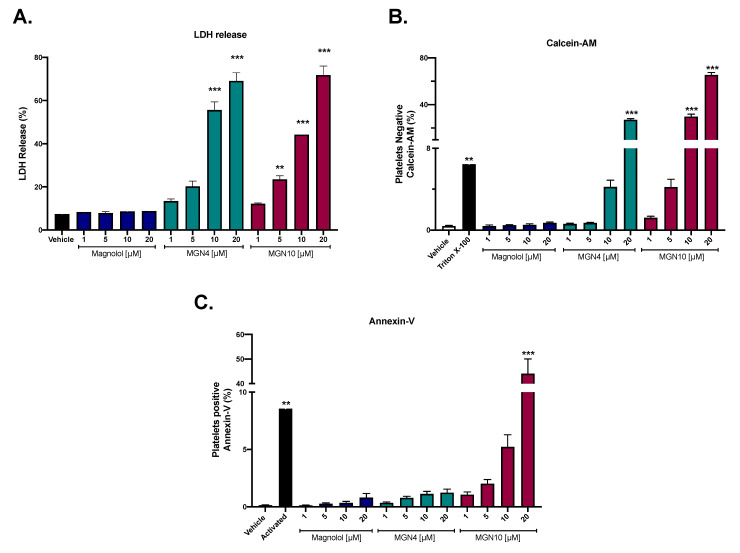
**Platelet cytotoxicity and apoptosis.** (**A**) LDH release from platelets. (**B**) Platelet viability by Calcein-AM. (**C**) Platelet apoptosis by Annexin-V. The results shown were obtained from at least five independent donors and expressed as the mean ± SEM. Vehicle: DMSO 0.4%. The statistical analysis was performed using a one-way analysis of variance (ANOVA) and the Bonferroni post hoc test. ** *p* < 0.01 and *** *p* < 0.001 vs. vehicle.

Figure 1 shows the cytotoxic effect of magnolol, MGN4, and MGN10 at concentrations ranging from 1 to 20 µM. LDH release analysis showed that magnolol does not produce cytotoxic effects on platelets, while MGN4 (10 and 20 µM) and MGN10 (5, 10, and 20 µM) showed a significant increase in the release of LDH from platelets, which is associated with an increased permeability or membrane damage that generates cytotoxicity (Figure 1A). Similar results were obtained with platelet viability by Calcein-AM since MGN4 (20 µM) and MGN10 (10 and 20 µM) significantly increased the percentage of non-viable platelets (Figure 1B). Finally, significant platelet apoptosis, measured by Annexin-V binding, was observed only at 20 µM MGN10 (Figure 1C).

### 2.2. Platelet Aggregation Results

**Figure 2:**
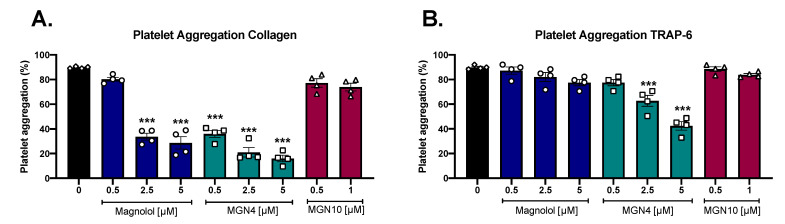
**Platelet aggregation results**. Platelet aggregation was induced by (**A**) collagen or (**B**) TRAP-6, as explained in the Methods. The aggregation assays were performed on washed platelets and the results are expressed as the percentage of aggregation after 5 min of reaction. Compounds were only tested at concentrations free of cytotoxicity. The results were obtained from at least four different donors and expressed as the mean ± SEM. Vehicle: DMSO 0.4%. The statistical analysis was performed using a one-way analysis of variance (ANOVA) and the Bonferroni post hoc test. *** *p* < 0.001 vs. vehicle.

Figure 2A,B show the effect on platelet aggregation of the three compounds when aggregation was triggered by collagen (2 µg/mL) and TRAP-6 (5 µM). A significant decrease in collagen-stimulated aggregation was observed in platelets preincubated with magnolol and MGN4. In the case of platelet aggregation stimulated by TRAP-6, only MGN4 (2.5 and 5 µM) produced a significant decrease. MGN10 was only tested at non-cytotoxic concentrations (0.5 and 1 µM) and did not present inhibitory effects on aggregation with any of the agonists tested. The inhibitory potency of the compounds was compared by obtaining the inhibitory concentration 50 (IC_50_), corresponding to the concentration at which aggregation is inhibited by 50%. The results are summarized in Table 1. The most potent compound was MGN4, as presented with the lowest IC_50_ values for both collagen and TRAP-6 (Table 1). The IC_50_ for collagen for MGN4 was approximately 3 times stronger when compared to the IC_50_ of magnolol (0.59 ± 0.3 µM vs 1.78 ± 0.6 µM, respectively). Similar results were obtained when TRAP-6 was the agonist, suggesting that the insertion of the triphenylphosphonium moiety (TPP+) into the magnolol structure to obtain MGN4 increased the antiplatelet effect of the compound.

### 2.3. Platelet Activation Markers

**Figure 3:**
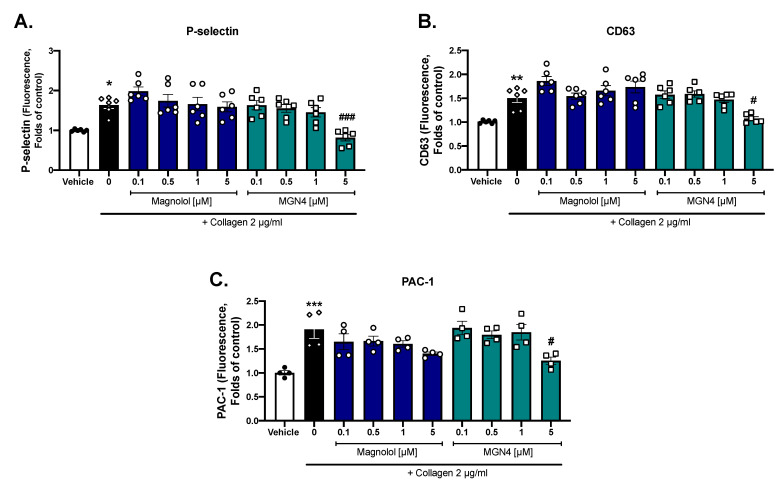
**Effect of magnolol and MGN4 on platelet activation markers.** (**A**) P-selectin expression. (**B**) CD63 expression. (**C**) GP IIb/IIIa activated (PAC-1) expression. Platelets were identified as the CD61 positive population and these CD61-expressing cells were analyzed in terms of change in mean fluorescence intensity than the vehicle. The results were obtained from at least four independent donors and bars represent the mean ± SEM. Vehicle: DMSO 0.4%. The statistical analysis was performed using a one-way analysis of variance (ANOVA) and the Bonferroni post hoc test. * *p* < 0.05 ** *p* < 0.01 and *** *p* < 0.001 vs vehicle; # *p* < 0.05 and ### *p* < 0.001 vs. activated control.

Considering the cytotoxicity of MGN10 and the absence of the inhibition of platelet aggregation at the concentration tested, we continued our studies comparing just magnolol and MGN4. As shown in Figure 3, we evaluated three platelet membrane markers that increased their expression after activation (P-selectin; CD63 and PAC-1). To compare the effect between magnolol and MGN4 against collagen stimulation, we used inhibiting concentrations above and below the IC_50_ obtained in aggregation (Table 1). MGN4 5 µM was able to significantly decrease the expression of the three markers evaluated when compared to the activated control, while magnolol did not show significant effects. In contrast to the effects observed with collagen, platelet activation was not modified when using TRAP-6 (data not shown), suggesting different mechanisms of action depending on the platelet agonist.

### 2.4. Effect on Mitochondrial Function

**Figure 4:**
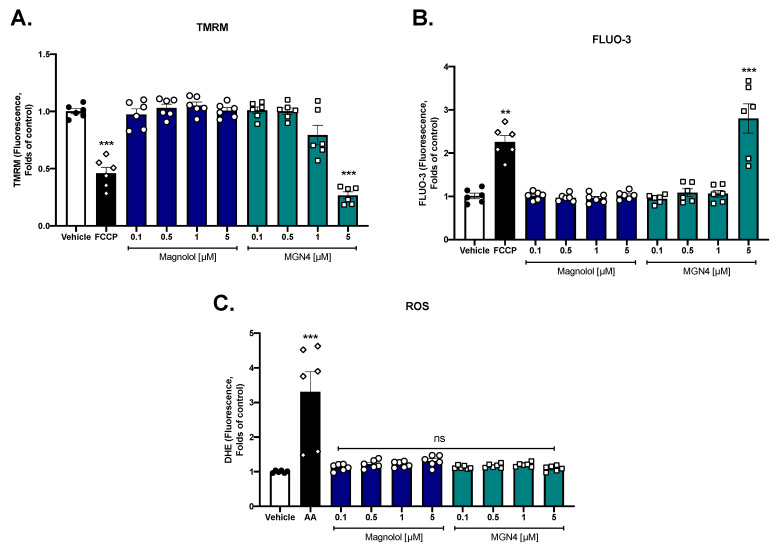
**Effect of magnolol and MGN4 on platelet mitochondria.** (**A**) Mitochondrial membrane potential (∆Ψm). (**B**) Intraplatelet calcium levels. (**C**) Intraplatelet ROS levels. The results were obtained by flow cytometry and platelets were identified as the CD61 positive population; these CD61-expressing cells were analyzed in terms of change in mean fluorescence intensity compared to the vehicle condition. FCCP was used as a positive control of mitochondrial depolarization and increased intraplatelet calcium levels; antimycin A (AA) was used as a positive control of ROS production. The results were obtained from at least six independent donors and expressed as the mean+ SEM. Vehicle: DMSO 0.4%. The statistical analysis was performed using a one-way analysis of variance (ANOVA) and the Bonferroni post hoc test. Non-statistical difference: ns, ** *p* < 0.01 and *** *p* < 0.001 vs. vehicle.

To see the effect of magnolol and MGN4 on the mitochondria, modifications of the mitochondrial membrane potential, calcium levels, and intracellular ROS were evaluated. MGN4 at 5 µM, similar to the positive control FCCP, significantly decreased the mitochondrial membrane potential in platelets (Figure 4A). Regarding the intracellular calcium levels, FCCP and MGN4 5 µM significantly increased the calcium signal (Figure 4B), which is probably associated with the fact that the decrease in mitochondrial membrane potential produces calcium release into the cytosol. In both cases, magnolol did not exert any effect (Figure 4A and Figure 4B). Both magnolol and MGN4 did not affect intraplatelet ROS levels and only the positive control (antimycin A) significantly increased the ROS levels (Figure 4C). The effect of Spautin-1 (a specific inhibitor of autophagy) on platelet aggregation can be seen in Figure S1. The results show that magnolol and MGN4 both at 2.5 and 5 µM, significantly decreased the aggregation of collagen-stimulated platelets. In the presence of Spautin-1, a significant reversal of the inhibition caused by MGN4 was observed at concentrations of 2.5 and 5 µM. This could be because the antiplatelet mechanism of MGN4 is associated with the activation of autophagy. In the case of magnolol, there was no significant reversal of the inhibitory effect on platelet aggregation in the presence of Spautin-1.

### 2.5. MGN4 Affects Mitochondrial Function in Platelets

**Figure 5:**
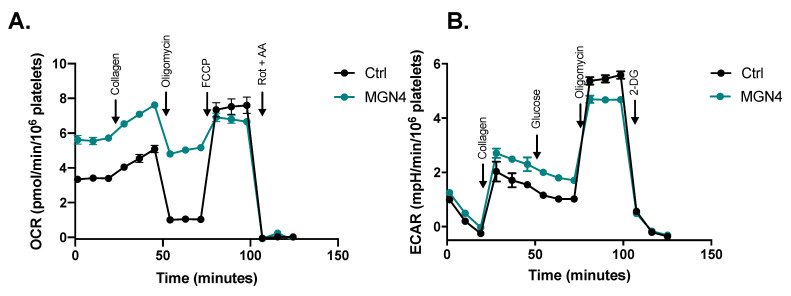
**Effect of MGN4 on platelet mitochondrial function.** (**A**) Representative profile of oxygen consumption rate (OCR) of platelets (25 × 10^6^ cells/well). OCR was measured in a Seahorse XFe24 extracellular flux analyzer (Agilent, Santa Clara, CA, US) before and after the sequential addition of 3 µg/mL collagen, 2.5 µM oligomycin, 1.4 µM FCCP, and 2 µM/2 µM rotenone/antimycin A. (**B**) Representative profile of extracellular acidification rate (ECAR) of platelets (25 × 10^6^ cells/well). ECAR was measured in a Seahorse XFe24 extracellular flux analyzer before and after the sequential addition of 3 µg/mL collagen, 10 mM glucose, 2.5 µM oligomycin, and 100 mM 2-DG. Data are the means ± SD (n ≥ 3). The experiment was repeated for two different donors and the results were similar (not shown).

Since mitochondrial membrane potential was modified by MGN4, we decided to further understand the mechanism of action of MGN4 in platelet metabolism. Thus, the platelet mitochondrial function when activated by collagen was studied by using a Seahorse extracellular flux analyzer (Figure 5A). Platelets exposed to 5 µM MGN4 showed several differences from the control including increased basal respiration, collagen-induced respiration, and ATP-independent respiration and reduced ATP-dependent respiration and non-mitochondrial respiration (Table 2). No differences were found in the activation and maximum respiratory capacity. Importantly, the control using BUFOS did not exert any effect on the respiration parameters of the activated platelets (Figure S2 and Table S1). Calculated coupling efficiency showed that MGN4-treated platelets were highly uncoupled, a result that agrees with the decreased mitochondrial membrane potential observed in Figure 4A. On the other hand, the analysis of ECAR on the MGN4-treated platelets showed an increase in glycolysis rate in parallel with a decreased ATP-dependent respiration (Figure 5B and Table 2). These effects were not detected when using the control BUFOS (Figure S2). In all of the tested conditions, the non-glycolytic acidification was similar (Table 2 and Table S1).

## 3. Discussion

Neolignans, specifically magnolol, have been widely investigated for their biological and pharmacological activities, for example, antitumor, anti-inflammatory, cardiovascular protection, antiangiogenesis, hypoglycemic, and antioxidant effects [42]. Due to its high therapeutic potential, clinical trials have been carried out for up to one year to test the possible side effects in humans after oral intake as a concentrated extract; no mutagenic potential, genotoxicity, and no significant adverse effects were reported in the participants [31]. Thus, we decided to evaluate the capacity of magnolol to exert antiplatelet effects, and in particular, we inserted a TPP+ group bonded by different carbon chain length linkers to direct the products to mitochondria. The antiplatelet effects of the compounds were not attributable to the cytotoxicity of magnolol and MGN4, at least up to 5 μM and 10 μM, as shown by the release of LDH and platelet viability by Calcein-AM, respectively. When conjugating magnolol with the TPP+ cation linked by a chain of 10 carbons (MGN10), cytotoxicity was present as low as 5 µM. These cytotoxicity data on platelets associated with the length of the chain were quite correlated with the results presented in the literature where other structures conjugated with the TPP+ cation and a 10-carbon chain linker were used (i.e., mitoquinone (MitoQ) [28], honokiol (HNK), lonidamine (LDN), and atovaquone (ATO) [43]). These results indicate that short-length carbon alkyl side chains can be used to conjugate molecules with a TPP+ cation and avoid platelet membrane distortion at low micromolar concentrations before showing cytotoxicity. Phosphatidylserine (PS) exposure quantified by Annexin-V binding is a marker of apoptosis in platelets [44], however, it can also regulate the procoagulant activity observed in platelet mitochondrial dysfunction [24]. In this context, only MGN10 at the highest concentration (20 µM) exhibited a significant apoptotic effect, while magnolol and MGN4, at neither of the concentrations tested, were shown to cause apoptosis.

Magnolol has been shown to have cardioprotective potential through antioxidant and vasodilator effects; it has been reported to be able to protect the heart from ischemic/reperfusion injury, reduce atherosclerosis, and inhibit neutrophil endothelial adhesion [41,45]. Specifically, in the endothelium, magnolol (20 µM) significantly suppressed the expression of platelet endothelial cell adhesion molecule (PECAM) and had effects at the mitochondrial level by increasing ROS, apoptosis, and the activation of caspase-3 cleaved [46]. The antiplatelet and antithrombotic effect of magnolol has also been described since it decreased the aggregation and secretion of ATP in platelet-rich plasma of mice stimulated with collagen and arachidonic acid; it also affected the increase in intracellular calcium and the formation of thromboxane B2 [47]. In rabbit platelets, magnolol (20–60 μM) inhibited platelet activation triggered by collagen-decreasing aggregation, calcium mobilization, and COX-1 activity by upregulating the PPAR-β/γ-dependent pathways [48]. Furthermore, it inhibited serotonin release [49].

In the current study, we demonstrated an antiplatelet effect of magnolol on collagen-activated human platelets by analyzing platelet aggregation. The addition of TPP+ with a four-carbon linker (MGN4) considerably enhanced the magnolol antiplatelet effect by a 3-fold decrease in the IC_50_ (0.59 ± 0.3 μM vs 1.78 ± 0.6 µM, respectively). Furthermore, only MGN4 inhibited the expression of the platelet activation markers P-selectin, CD63, and PAC-1, in contrast to what was observed for magnolol. Moreover, MGN4 was also able to show a small effect on the protease-activated receptor pathway, stimulated by TRAP-6.

Among the most important biological effects shown by magnolol is its antitumor action as it can inhibit the proliferation, migration, and invasion in vivo and/or in vitro of pancreatic cancer [50], some sarcomas [51] and carcinomas [52]. The antitumor mechanisms of magnolol are associated with the activation of apoptosis through increased expression of proapoptotic proteins (Bid, Bax, and cytochrome c), mitochondrial pore opening, increased intracellular ROS and decreased ∆Ψm, which trigger mitochondrial dysfunction and autophagy/mitophagy processes [53].

In the case of platelets, we observed that the use of Spautin-1 (inhibitor of autophagy) showed a reversal of the inhibition caused by MGN4 on platelet aggregation, which can be explained by the need for a high-level basal rate of autophagy for platelet activation, aggregation, hemostasis, and thrombosis [54,55].

The literature suggests that magnolol has direct effects on mitochondria, but at the concentrations used under our experimental conditions on human platelets (0.1–5 μM), no effects on ∆Ψm, intracellular calcium levels, and ROS were observed. In contrast, MGN4 significantly decreased ∆Ψm and increased intraplatelet calcium (product of mitochondrial dysfunction) in a dose-dependent manner, which is associated with the accumulation of MGN4 in platelet mitochondria. This accumulation can be ascribed to the addition of the cation TPP+ into magnolol, forming MGN4, which could be the cause of the higher antiplatelet potential of MGN4.

The group TPP+ has been extensively used to send different compounds specifically to the mitochondria [20]. When performing mitochondrial function studies, we observed that MGN4 affects platelet activation and generates changes in ∆Ψm, which can be caused by the mitochondria of the uncoupled platelets. This was shown when platelets preincubated with MGN4 presented an increased basal oxygen consumption rate, indicating that the absence of an aggregation stimulus by MGN4 is the product of uncoupling the platelets’ mitochondria caused by this compound. Importantly, this effect was not observed when using the control compound BUFOS, supporting that the linking of the TPP+ moiety to the magnolol generates respiration uncoupling. Moreover, in the presence of MGN4, ATP-dependent respiration was decreased in addition to changes in ECAR. The increase in the glycolytic rate supports a metabolic shift of the glycolytic pathway due to MGN4. If undesired mitochondrial dysfunction occurs in platelets, non-desired activation of the platelets occurs with an increase in platelet activation and aggregation, leading to changes in their capacity to aggregate and coagulate when needed. Thus, the former process must be controlled when using antiplatelet drugs for the long-term. Overall, our data confirm that the addition of the TPP+ moiety to Magnolol improves its protective effects, potentiating the antiplatelet capacity of this compound, as well as showing that mitochondria are the target of MGN4. This research can be used as an antecedent to develop novel antiplatelet agents derived from natural products.

## 4. Materials and Methods

### 4.1. Chemical Structure of Compounds

**Figure 6:**
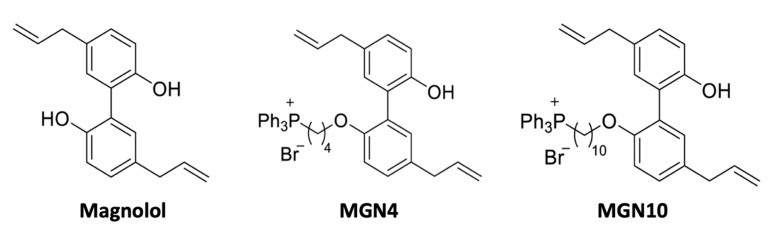
**Chemical structure of compounds.** MGN4 and MGN10 refer to a TPP+ moiety conjugated to MGN (Magnolol) via a 4- and 10-carbon alkyl side chain, respectively [30].

MGN4 and MGN10 refer to a TPP+ moiety conjugated to magnolol (MGN) via a 4- and 10-carbon alkyl side chain, respectively, as shown in Figure 6 [30]. These compounds were facilitated by Dr. Balaraman Kalyanaraman at the Medical College of Wisconsin, USA.

### 4.2. Purification of Washed Human Platelets

Venous phlebotomy was performed on voluntary donors (10 days without medication) who agreed to participate in the study through informed consent (protocol approved by the Scientific Ethics Committee of the University of Talca, No. 04-2022) [56]. For the extraction, acid citrate dextrose (ACD) was used, which was mixed with the whole blood in a ratio of 4:1 *v/v*. The blood was centrifuged at room temperature (RT) for 12 min at 250× *g* to obtain platelet-rich plasma (PRP). The extracted PRP was centrifuged for 8 min at 900× *g* to precipitate the platelets. The supernatant was removed and the platelet pellet was suspended in Tyrodes buffer without calcium plus ACD at a ratio of 5:1 *v*/*v*. Platelets were again centrifuged for 8 min at 900 g. Finally, the platelet pellet was resuspended in a Tyrodes buffer without calcium. The final concentration of platelets for each experiment was adjusted with the Mindray BC-3000 Plus hematology counter, Japan [57].

### 4.3. Cytotoxic Activity by LDH Release

Washed platelets (200–250 × 10^6^ platelets/mL) were incubated with magnolol, MGN4, or MGN10 (1, 5, 10, and 20 µM) for 10 min at 37 °C. DMSO was used as the vehicle. Platelets were then centrifuged at 900× *g* for 8 min to obtain the supernatant, which was mixed with the working reagent of the Lactate Dehydrogenase (LDH) Cytotoxicity Kit (Cayman Chemical, Ann Arbor, MI, USA). The maximum cytotoxicity control corresponds to Triton X-100 at 10% [58].

### 4.4. Cell Viability by Calcein-AM

Washed platelets (200–250 × 10^6^ platelets/mL) were labeled with Calcein-AM and incubated for 20 min at 37 °C in the dark. Subsequently, the compounds under study (1, 5, 10, and 20 µM) were added and incubated for 10 min at 37 °C in the dark. DMSO was used as the vehicle. Subsequently, the population of CD61+ platelets was identified, and their viability was determined with the BD FacsLyric flow cytometer (BD Biosciences, San Diego, CA, USA). The fraction (%) of calcein-negative platelets in the CD61-positive subpopulation was recognized as non-viable platelets. Triton X-100 0.1% was used as the cell damage control [28].

### 4.5. Apoptosis Activity (Externalization of Phosphatidylserine)

Washed platelets (200–250 × 10^6^ platelets/mL) were incubated with magnolol, MGN4, or MGN10 (1, 5, 10, and 20 µM) for 10 min at 37 °C. DMSO was used as the vehicle. An aliquot from each condition was incubated with Annexin-V FITC to identify increased externalization of phosphatidylserine (PS) with the BD Facs Lyric flow cytometer (Annexin V-FITC Apoptosis Detection/Staining Kit, ABCAM, Boston, MA, USA). The apoptotic platelet population was identified as the fraction (%) of Annexin V positive platelets. As a positive control of procoagulant/apoptosis activity, hyperactivated platelets were used with a mixture of collagen (2 µg/mL) and TRAP-6 (10 µM) [28].

### 4.6. Platelet Aggregation

Briefly, platelet aggregation was evaluated in washed platelets (200–250 × 10^6^ platelets/mL) in an aggregometer AggRAM Analyzer (Helena Laboratories, Beaumont, TX, USA). Washed platelets (with 2 mM CaCl_2_) were preincubated with the compounds for 5 min at 37 °C inside the aggregometer. Then, aggregation was initiated with either collagen (2 µg/mL) or TRAP-6 (5 µM). The aggregation reaction was measured for 5 min at 37 °C under continuous stirring (1000 rpm) [59].

### 4.7. Platelet Activation Markers

Washed platelets (200–250 × 10^6^ platelets/mL) were preincubated with the compounds for 5 min at 37 °C and then activated with 2 µg/mL collagen followed by 5 min of incubation at 37 °C. Subsequently, aliquots were taken and labeled separately with each of the antibodies against the activation markers P-selectin, CD63, and activated GPIIb/IIIa (PAC-1). The reading was performed on a BD FacsLyric flow cytometer (BD Biosciences, San José, CA, USA). CD61 FITC was used to identify the platelet population [58,60].

### 4.8. Mitochondrial Membrane Potential (∆*Ψ*m)

Washed platelets (50 × 10^6^ platelets/mL) were labeled with the 100 nM tetramethylrhodamine methyl ester perchlorate (TMRM) potentiometric probe and incubated for 20 min at 37 °C in the dark. They then added the different concentrations of the compounds under study and incubated them for 10 min at 37 °C in the dark. The reading was performed on a BD FacsLyric flow cytometer (BD Biosciences, San José, CA, USA). The mitochondrial depolarization control used was carbonyl cyanide-p-trifluoromethoxyphenylhydrazone (FCCP) 1 µM [57]

### 4.9. Intraplatelet ROS Levels

Reactive oxygen species (ROS) levels were determined in washed platelets (5 × 10^6^ platelets/mL) using dihydroethidium (DHE, 10 µM). The platelets were incubated with DHE for 20 min at 37 °C in the dark followed by another 10 min of incubation with different concentrations of the compounds. The reading was performed on the BD FacsLyric flow cytometer (BD Biosciences, San José, CA, USA). Antimycin A 20 µM was used as a positive control for ROS increase [43].

### 4.10. Intraplatelet Calcium Levels

Briefly, washed platelets (200–250 × 10^6^ platelets/mL) were labeled with Fluo-3-AM (0.44 µM) and incubated for 30 min at room temperature in the dark. Subsequently, they were diluted to a count of 50 × 10^6^ platelets/mL and incubated for 10 min at 37 °C with the different concentrations of the compounds under study. The reading was performed on a BD FacsLyric Flow Cytometer (BD Biosciences, San José, CA, USA). P-trifluoromethoxyphenylhydrazone carbonylcyanide (FCCP) 1 µM was used as a positive control for the increase in intracellular calcium [43].

### 4.11. Oxygen Consumption Rate and Extracellular Acidification Rate Assays

Oxygen consumption rate (OCR) and extracellular acidification rate (ECAR) were measured using a Seahorse XFe24 Extracellular Flux Analyzer (Agilent, Santa Clara, CA, US). Washed platelets were seeded (20–25 × 10^6^ cells/well) in 100 µL of modified Tyrode’s-HEPES buffer and then centrifuged at 300× *g* for 10 min without a break to allow for the adhesion of the cells to the plate. Platelets were incubated at 25 °C for 5 min with 5 µM MGN4 in the same buffer (final volume 600 µL). Then, Tyrode’s-HEPES buffer was removed and Seahorse medium (8.3 g/L DMEM, 1.85 g/L NaCl, 5 mM glucose, 1 mM pyruvate, 2 mM glutamine, 5 mM HEPES, pH 7.4) was added to a final volume of 600 µL [61]. As a control, the butyltriphenylphosphonium bromide (BUFOS), which is the TPP+ moiety linked to the 4-carbon alkyl chain, was used at 5 µM. The oxygen consumption rate was measured before and after the sequential addition of 3 µg/mL collagen, 2.5 µM oligomycin, 1.4 µM FCCP, and 2 µM/2 µM antimycin A/rotenone. The non-mitochondrial oxygen consumption rate (obtained after the addition of antimycin A/rotenone) was subtracted from all measurements. Respiratory parameters were obtained as follows: Basal (baseline OCR), collagen (OCR after the addition of collagen), activation (collagen-basal), ATP-independent (OCR resistant to the addition of oligomycin), ATP-dependent (basal-ATP-independent), maximum (OCR obtained after the addition of FCCP), and spare (maximum-basal) [62,63]. Platelet respiration was normalized considering the cell number determined with the automated cell counter Z1 Coulter Particle Counter (Beckman, Indianapolis, IN, USA).

### 4.12. Statistical Analysis

Data were analyzed with Prism 8.0 software (GraphPad Inc., San Diego CA, USA) and expressed as the mean ± standard error of the mean (SEM). Differences between groups were analyzed using a one-way analysis of variance (ANOVA) and Bonferroni’s post hoc test, unless stated otherwise. *p* values < 0.05 were considered statistically significant.

## Figures and Tables

**Table 1:** IC50 values obtained for each compound and agonist.

Compound	IC_50_ Collagen [μM]	IC_50_ TRAP-6 [μM]
Magnolol	1.78 ± 0.6	>20 *
MGN4	0.59 ± 0.3	13.94 ± 6.65 *
MGN10	>5 *	>20 *

* cytotoxic concentration as indicated in Figure 1.

**Table 2:** Quantification of respiration and acidification rate parameters.

Rate	Control	MGN4 (5 µM)
**Basal** (OCR/10^6^ platelets)	3.4 (0.2)	5.6 (0.6) ****
**Collagen** (OCR/10^6^ platelets)	4.6 (0.3)	7.1 (0.3) ****
**Activation** (OCR_Collagen_—OCR_Basal_)	1.2 (0.4)	1.5 (0.3)
**ATP-indep** (OCR/10^6^ platelets)	1.0 (0.2)	5.0 (0.4) ****
**ATP-dep** (OCR/10^6^ platelets)	2.4 (0.1)	0.6 (0.4) ****
**Maximum** (OCR/10^6^ platelets)	7.5 (0.8)	6.8 (0.6)
**Spare** (OCR_Maximum_—OCR_Basal_)	4.1 (1.0)	1.2 (0.8) ****
**Non-mito** (OCR/10^6^ platelets)	3 (0.4)	0.9 (0.5) ****
**Coupling efficiency** ((OCR_Basal_—OCR_ATP-indep_)/OCR_Basal_)	0.70 (0.03)	0.11 (0.06) **
**Glycolysis** (mpH/min/10^6^ platelets)	1.1 (0.1)	1.8 (0.2) ***
**Glycolytic capacity** (mpH/min/10^6^ platelets)	5.5 (0.2)	4.7 (0.1) ***
**Non-glycolytic acidification** (mpH/min /10^6^ platelets)	1.3 (0.1)	1.5 (0.1)

Means (SD), n ≥ 3 of the respiratory and glycolytic parameters. The value for the non-mitochondrial respiration rate and non-glycolytic acidification rate in each well was subtracted from all other values. Significance was tested using two-way ANOVA with Sidak’s multiple comparisons tests or the unpaired Student’s *t*-test. ** *p* < 0.01, *** *p* < 0.001, **** *p* < 0.0001.

## Data Availability

Data is contained within the article and in Supplementary Materials.

## Supplementary Materials

The following supporting information can be downloaded at https://www.mdpi.com/article/10.3390/ph16020210/s1: Figure S1. Effect of Spautin-1 on platelet aggregation; Figure S2. Determination and quantification of respiration and acidification rate parameters of BUFOS; Table S1. Quantification of respiration and acidification rate parameters of BUFOS.
