# Supplementary material for: The Use of Triphenyl Phosphonium Cation Enhances the Mitochondrial Antiplatelet Effect of the Compound Magnolol
Source: Pharmaceuticals (Basel). 2023 Jan 30;16(2):210. doi: 10.3390/ph16020210 (PMC9958981; doi:10.3390/ph16020210)
Supplement: Supplementary file 1 [file pharmaceuticals-16-00210-s001.zip › pharmaceuticals-2001804-supplementary.pdf]

Supplementary data

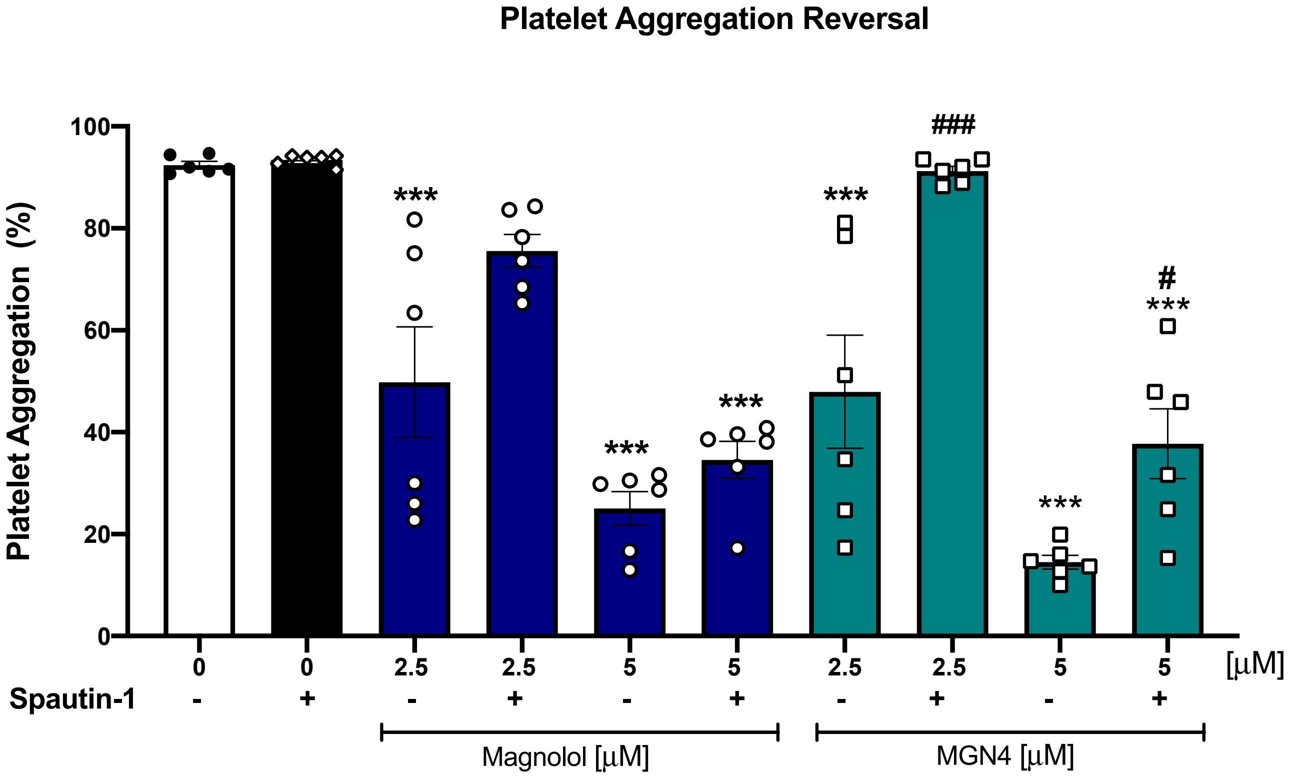

Figure S1. Effect of Spautin-1 on platelet aggregation. The aggregation assays were performed on washed platelets and the results are expressed as the percentage of aggregation after 5 minutes of preincubation with Spautin-1 and/or Magnolol or MGN4 followed by 5 minutes of reaction. Compounds were only tested at concentrations free of cytotoxicity. The results are expressed as mean ± SEM (*n*=6). Vehicle: DMSO 0.4%. The statistical analysis was performed using a one-way analysis of variance (ANOVA) and the Bonferroni post hoc test. ***p<0.001 vs vehicle (with and without Spautin-1). #p<0.05 and ###p<0.001 vs same concentration compound without Spautin-1

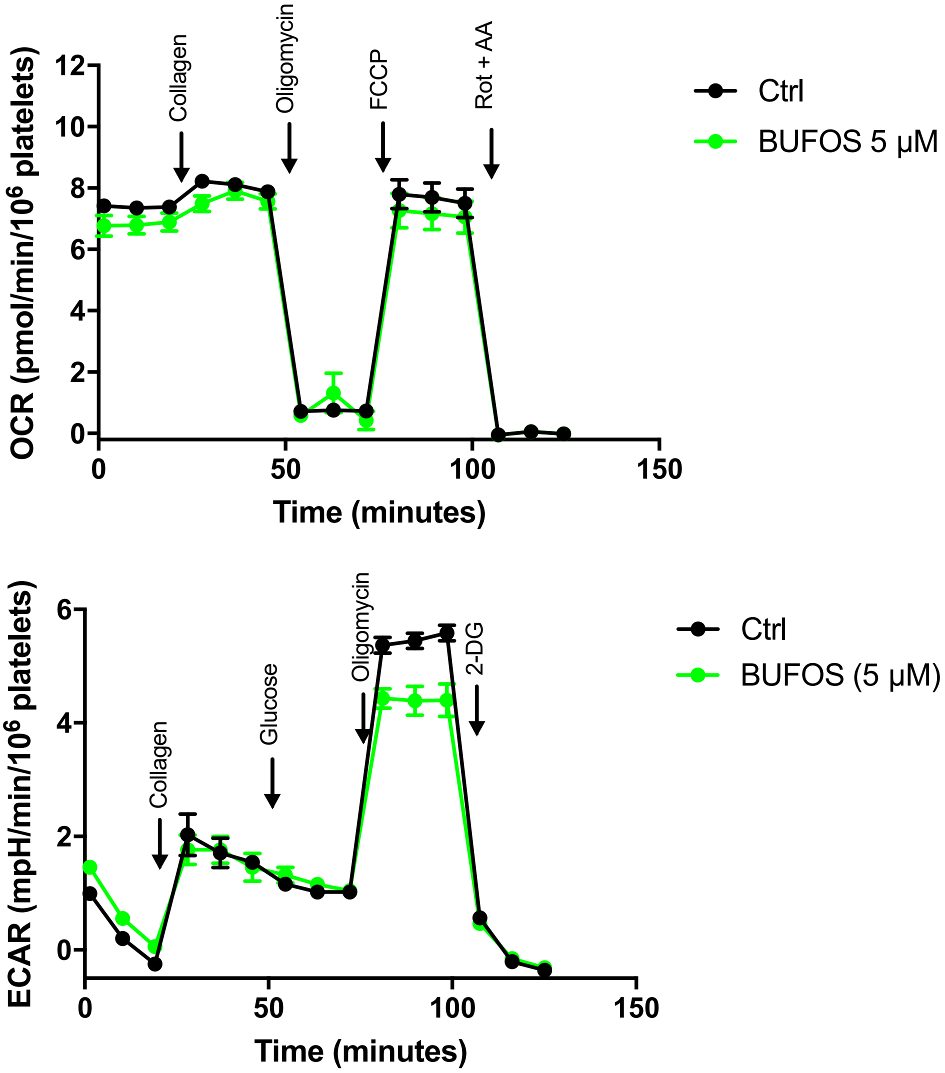

Figure S2. Determination and quantification of respiration and acidification rate parameters of BUFOS. The respiration and acidification experiments were done under the same experimental conditions as in figure 6 of the manuscript using bufos (5 μM) as a control of the effects of the TPP+ moiety. Significance was tested using two-way ANOVA with Dunnet's multiple comparisons test or unpaired Student's t-test. **** P < 0.0001.

Table S1. Quantification of respiration and acidification rate parameters of BUFOS.

Rate	Control	BUFOS (5 μM)
**Basal** (OCR/10^6 platelets)	7.4 (0.1)	6.8 (0.6)
**Collagen** (OCR/10^6 platelets)	8.4 (0.2)	8.0 (0.5)
**Activation** ($OCR_{Collagen} - OCR_{Basal}$)	1.0 (0.1)	1.2 (0.5)
**ATP-indep** (OCR/10^6 platelets)	0.7 (0.1)	0.8 (0.5)
**ATP-dep** (OCR/10^6 platelets)	6.6 (0.2)	6.0 (0.2)
**Maximum** (OCR/10^6 platelets)	7.7 (0.8)	7.2 (1.1)
**Spare** ($OCR_{Maximum} - OCR_{Basal}$)	0.3 (0.8)	0.3 (0.9)
**Non-mito** (OCR/10^6 platelets)	2.0 (0.8)	1.9 (0.6)
**Coupling efficiency** (($OCR_{Basal} - OCR_{ATP\text{-}indep}$)/$OCR_{Basal}$)	0.9 (0.1)	0.9 (0.1)
**Glycolysis** (mpH/min/10^6 platelets)	1.1 (0.1)	1,2 (0.1)
**Glycolytic capacity** (mpH/min/10^6 platelets)	5.5 (0.2)	4,4 (0.4) ****
**Non-glycolytic acidification** (mpH/min /10^6 platelets)	1.3 (0.1)	1,1 (0.1)

Means (SD), n ≥ 3 of respiration and glycolytic parameters. The value for the non-mitochondrial respiration rate and non-glycolytic acidification rate in each well was subtracted from all other values. Significance was tested using two-way ANOVA with Sidak's multiple comparisons tests or unpaired Student's t-test. ** P < 0.01, *** P < 0.001, **** P < 0.0001.
